# Microwave Application and Anhydrous Cu(OAc)_2_ Mediated O-Arylation of Aliphatic Amino Alcohols

**DOI:** 10.4236/ijoc.2016.62011

**Published:** 2016-05-27

**Authors:** Mohammad Al-Masum, Linda Quinones, Laurance T. Cain

**Affiliations:** Department of Chemistry, Tennessee State University, Nashville, TN, USA

**Keywords:** Hydroxylamine, Amino Ether, O-Arylation, O-Styrylation, Microwave

## Abstract

Anhydrous Cu(OAc)_2_ mediated efficient protocol has been developed in the area of C-O coupling from potassium aryltrifluoroborates and aliphatic amino alcohols such as β-hydroxy, γ-hydroxy, and δ-hydroxy amines. The scope of this transformation focuses on direct O-arylation and Ostyrylation. The reaction vial loaded with reactants under argon atmosphere is microwaved at 140°C for 30 min to furnish the corresponding cross-coupling product, amino ethers, in good yields.

## 1. Introduction

**Scheme 1:**
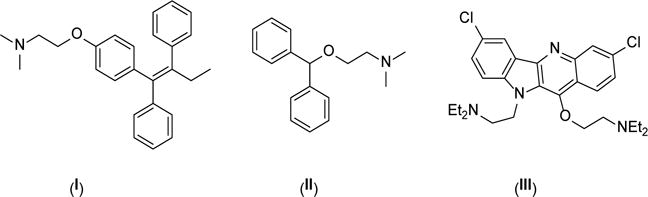
Amino ethers.

Amino ethers are important intermediates in organic synthesis and compounds of pharmaceutical interest such as tamoxifen **(I)**, antihistamines **(II)**, potent marine natural products such as quindolone **(III)**, and also agricultural interest such as water-based organic coating amino ether surfactants [1]–[7] (Scheme 1).

Potassium organotrifluoroborates have already been proven as effective organoboron reagents in cross-coupling chemistry [8]–[10]. Recently, this reagent is used in copper-promoted carbon-oxygen cross-coupling reaction. Batey’s group has reported a protocol for the alkyl-aryl and alkyl-vinyl ethers via Cu (II)-catalyzed cross-coupling of organotrifluoroborates and aliphatic alcohols [11]–[17]. Chan [18]–[20] and Lam’s groups reported heteroatom arylation reaction for alkyl-aryl ether synthesis although this observation was limited to phenols only. Further development of copper-mediated C–O bond formation has explained by oxygen nucleophiles such as carboxylic acids, aliphatic alcohols, aryl oximes, silanols, N-hydroxypthalimides, water with boron reagents [21]–[23].

But using aliphatic hydroxyl amine for similar cross-coupling reaction and making amino ether are rarely known. Very recently, Molander’s group [24]–[27] reported an effective protocol toward the O-arylation of *β*-hydroxy-*α*-amino acid substrates. Molander’s report of O-arylation of protected serines and threonines by introducing amino alcohols, such as *β*-hydroxy-*α*-amino acid derivatives with arylboronic acids and aryltrifluoroborates for the formation of C-O alkyl aryl ethers, is a new development of Chan-Lam cross-coupling process [24].

**Scheme 2:**
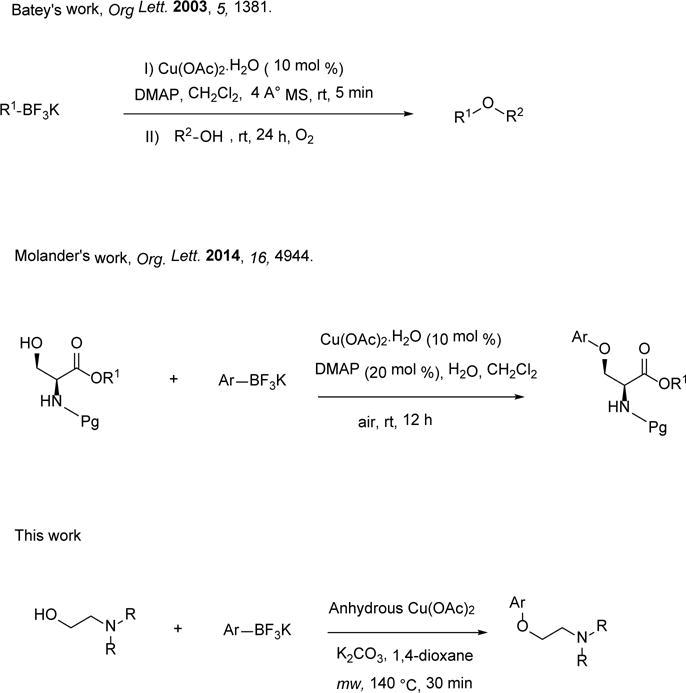
O-Arylation of alcohols and amino alcohols.

In this work, we also wanted to see whether anhydrous Cu(OAc)_2_ would be able to provide similar transformation in minutes under microwave irradiation and in the absence of air. Interest in exploring various organic transformations by using potassium organotrifluoroborates led to investigate the cross-coupling reaction of *β*-hydroxy, *γ*-hydroxy, and *δ*-hydroxy amines with potassium aryltrifluoroborates in the presence of anhydrous Cu(OAc)_2_ under microwave irradiation (Scheme 2). The C-O cross-coupling initiated with the optimization of the reaction partners and conditions for the formation of O-arylated amino ether moiety. We first investigated the catalytic activities of anhydrous Cu(OAc)_2_ (10 mole%, 20 mol%, and 50 mol%). No significant improvement was observed. Longer reaction time for more than 30 minutes and conventional heating system has no effect on increasing the yield. Other catalyst system such as palladium-catalyst was also employed and showed no product. Then we promote the model reaction of *β*-hydroxyamine such as 2-dimethylaminoethanol, 2a (1 equivalent), potassium tolyltrifluoroborate, **1a** (2.5 equivalent), K_2_CO_3_ (2.0 equivalent), and anhydrous Cu(OAc)_2_ (1 equivalent) in 2.0 mL 1,4-dioxane microwaved at 140°C for 30 minutes (Entry 1, Table 1). After chromatography 76% isolated amino ether product, **3a** was obtained. The product was characterized by GC/MS (Saturn 2200 Benchtop GC/MS) and NMR (Varian 300 MHz). GCMS: Calculated for C_11_H_17_NO M^+^ 180. Found: 180. ^1^H NMR (Acetone-d_6_, 300 MHz) *δ* 7.11 (d, J = 8.4 Hz, 2H, aromatic), 6.90 (d, J = 8.7 Hz, 2H, aromatic), 4.46 (t, J = 4.8 Hz, 2H, CH_2_), 3.85 (t, J = 4.8 Hz, 2H, CH_2_), 3.23 (s, 6H, 2 × CH_3_), 2.25 (s, 3H, CH_3_); ^13^C NMR (Ace-tone-d_6_, 75.5 MHz) *δ* 129.9, 114.5, 61.9, 56.8, 43.4, 19.5.

*γ*-hydroxy amine such as 3-diethylamino-1-propanol, **2b** and *δ*-hydroxyamine such as 4-(dimethylamino)-1-butanol, **2c** were used with tolyltrifluoroborate under the same reaction conditions afforded the corresponding amino ethers **3b** and **3c** in good yields (Entries 2 and 3, Table 1). In several other instances, amino alcohols **2a**, **2b**, **2c** are microwaved with various aryltrifluoroborates such as phenyltrifluoroborate, **1b**, 4-fluorophenyltrif-luoroborate, **1c**, 4-trifluoromethylphenyltrifluoroborate, **1d**, 4-trifluoromethoxyphenyltrifluoroborate, **1e**, and 4-chlorophenyltrifluoroborate, **1f**, in the presence of anhydrous Cu(OAc)_2_. In all cases, amino ether products were furnished (Products **3d-3k**, Table 1).

To explore the generality and scope of the O-arylation of *β*-hydroxy and *γ*-hydroxy amines, we examined the reaction with styryltrifluoroborates under the same reaction conditions. It worked well as shown in Table 2. In all cases, reaction looked very clean with *trans* selectivity. When subjected to silica gel chromatography, product didn’t collect effectively and showed less than expected yield.

Cu(OAc)_2_ mediated cross-coupling reaction of O-arylation typically requires air in the system for REDOX process. But, O-arylation of amino alcohols in the presence of anhydrous Cu(OAc)_2_ reported herein is completed under argon atmosphere, not in air. Excess K_2_CO_3_ may favor the transmetallation followed by reductive coupling and form the amino ether product.

In addition to Molander’s effective protocol toward copper(II)-mediated O-arylation of protected serines and threonines via Chan-Lam cross-coupling, this work of anhydrous copper acetate mediated reaction O-arylation and O-styrylation of amino alcohols for new series of aminoethers synthesis is interesting development.

## 2. Procedure

The product N, N-dimethyl-2-(*p*-tolyloxy) ethan-1-amine, **3a** from the cross-coupling of potassium tolyltrifluoroborate, **1a** and 2-dimethylaminoethanol, **2a** is shown as a representative procedure. The reaction was performed on a 0.5 mmol scale. After purging with argon, a microwave reaction tube with a stirrer bar was loaded with 246.0 mg (1.25 mmol) of potassium tolyltrifluoroborate, 138.0 mg (1.0 mmol) of K_2_CO_3_, 90.8 mg (0.5 mmol) of anhydrous Cu(OAc)_2_, and 50 μL (0.5 mmol) of 2-dimethylaminoethanol. The reaction tube was capped and flushed with argon followed by adding 2.0 mL of 1,4-dioxane. The resulting reaction mixture was then inserted in the microwave vessel (CEM Explorer 24, Discover SP, and 300 W) and irradiated at 140°C for 30 min. The crude reaction product was extracted from inorganic material using ethyl acetate followed by washing with brine and dried over anhydrous sodium sulphate. For purification the crude product was subjected to preparative TLC using hexane/ethyl acetate (2/1) as eluent and collected the 68.4 mg (76%) amino ether **3a**. The product was characterized by GC/MS (Saturn 2200 Benchtop GC/MS) and NMR (Varian 300 MHz).

### Compound **3a**

GCMS: Calculated for C_11_H_17_NO M^+^ 180. Found: 180. ^1^H NMR (Acetone-d_6_, 300 MHz) *δ* 7.11 (d, J = 8.4 Hz, 2H, aromatic), 6.90 (d, J = 8.7 Hz, 2H, aromatic), 4.46 (t, J = 4.8 Hz, 2H, CH_2_), 3.85 (t, J = 4.8 Hz, 2H, CH_2_), 3.23 (s, 6H, 2 × CH_3_), 2.25 (s, 3H, CH_3_); ^13^C NMR (Acetone-d_6_, 75.5 MHz) *δ* 129.9, 114.5, 61.9, 56.8, 43.4, 19.5.

### Compound **3b**

GCMS: Calculated for C_14_H_23_NO M^+^ 222. Found: 222. ^1^H NMR (Acetone-d_6_, 300 MHz) *δ* 7.05 (d, J = 8.7 Hz, 2H, aromatic), 6.82 (d, J = 8.4 Hz, 2H, aromatic), 4.13 (t, J = 5.7 Hz, 2H, CH_2_), 3.52 (m, 4H, 2 × CH_2_), 3.4 (t, J = 7.5 Hz, 2H, CH_2_), 2.24 (m, 2H, CH_2_), 1.4 (t, J = 7.2 Hz, 6H, 2 × CH_3_); ^13^C NMR (Acetone-d_6_, 75.5 MHz) *δ* 130.7, 115.2, 65.8, 50.7, 48.7, 24.9, 20.5, 9.4.

### Compound **3c**

GCMS: Calculated for C_13_H_21_NO M^+^ 208. Found: 208. ^1^H NMR (Acetone-d6, 300 MHz) *δ* 7.08 (d, J = 8.7 Hz, 2H, aromatic), 6.80 (d, J = 8.4 Hz, 2H, aromatic), 3.97 (t, J = 5.7 Hz, 2H, CH_2_), 2.60 (m, 2H, CH_2_), 2.42 (s, 6H, 2 × CH_3_), 2.23 (s, 2H, CH_2_), 1.78 (m, 4H, 2 × CH_2_); ^13^C NMR (Acetone-d_6_, 75.5 MHz) *δ* 130.6, 115.1, 68.2, 45.1, 27.6, 24.2, 20.5.

### Compound **3d**

GCMS: Calculated for C_10_H_15_NO M^+^ 166. Found: 166. ^1^H NMR (Acetone-d_6_, 300 MHz) *δ* 7.27 (m, 2H, aromatic), 6.92 (m, 3H, aromatic), 4.07 (t, J = 6.0 Hz, 2H, CH_2_), 2.67 (t, J = 6.0 Hz, 2H, CH_2_), 2.26 (s, 6H, 2 × CH_3_); ^13^C NMR (Acetone-d_6_, 75.5 MHz) *δ* 159.9, 130.3, 121.3, 115.3, 67.0, 59.0, 46.2.

### Compound **3e**

GCMS: Calculated for C_13_H_21_NO M^+^ 208. Found: 208. ^1^H NMR (Acetone-d6, 300 MHz) *δ* 7.26 (m, 2H, aromatic), 6.91 (m, 3H, aromatic), 4.05 (t, J = 6.3 Hz, 2H, CH_2_), 2.67 (t, J = 6.6 Hz, 2H, CH_2_), 2.57 (q, J = 7.2 Hz, 4H, 2 × CH_2_), 1.92 (m, 2H, CH_2_), a.03 (t, J = 7.2 Hz, 6H, 2 × CH_3_); ^13^C NMR (Acetone-d_6_, 75.5 MHz) *δ* 160.1, 130.3, 121.2 115.3, 66.5, 50.0, 47.8, 27.7, 12.1.

### Compound **3f**

GCMS: Calculated for C_10_H_14_NOF M^+^ 184. Found: 184. ^1^H NMR (Acetone-d_6_, 300 MHz) *δ* 7.0 (m, 4H, aromatic), 4.07 (m, 2H, CH_2_), 2.73 (t, J = 5.86, 2H, CH_2_), 2.31 (s, 6H, 2 × CH_3_); ^13^C NMR (Acetone-d_6_, 75.5 MHz) *δ* 116.6, 116.3, 67.6, 60.6, 58.8, 46.0, 20.8, 14.5.; ^19^F NMR (Acetone-d_6_, 300 MHz) *δ*–125.8.

### Compound **3g**

GCMS: Calculated for C_13_H_20_NOF M^+^ 225. Found: 225. ^1^H NMR (Acetone-d_6_, 300 MHz) *δ* 6.98 (m, 4H, aromatic), 4.0 (t, J = 6.0 Hz, 2H, CH_2_), 2.60 (d, J = 6.9 Hz, 2H, CH_2_), 4H, CH_2_), 2.53 (q, J = 7.2 Hz, 4H, 2 × CH_2_), 1.87 (m, 2H, CH_2_), 0.99 (t, J = 6.9 Hz, 6H, 2 × CH_3_); ^13^C NMR (Acetone-d_6_, 75.5 MHz) *δ* 116.6, 116.3, 67.3, 49.9, 47.7, 27.8, 12.3.

### Compound **3h**

GCMS: Calculated for C_11_H_14_NOF_3_ M^+^ 234. Found: 234. ^1^H NMR (Acetone-d_6_, 300 MHz) *δ* 7.62 (d, J = 8.4 Hz, 2H, aromatic), 7.12 (d, J = 8.7 Hz, 2H, aromatic), 4.17 (t, J = 5.7 Hz, 2H, CH_2_), 2.70 (t, J = 6.0 Hz, 2H, CH_2_), 2.27 (s, 6H, 2 × CH_3_); ^13^C NMR (Acetone-d_6_, 75.5 MHz) *δ* 127.8, 127.7, 115.7, 67.5, 58.7, 46.1; ^19^F NMR (Acetone-d_6_, 300 MHz) *δ*–61.8.

### Compound **3i**

GCMS: Calculated for C_11_H_14_NO_2_F_3_ M^+^ 250. Found: 250. ^1^H NMR (Acetone-d_6_, 300 MHz) *δ* 7.23 (d, J = 9.2 Hz, 2H, aromatic), 7.03 (d, J = 9.3 Hz, 2H, aromatic), 4.12 (t, J = 6.0 Hz, 2H, CH_2_), 2.73 (m, 2H, CH2), 2.30 (s, 6H, 2 × CH_3_); ^13^C NMR (Acetone-d_6_, 75.5 MHz) *δ* 158.8, 129.7, 123.4, 116.4, 115.4, 67.5, 58.8,46.1; ^19^F NMR (Acetone-d_6_, 300 MHz) *δ* 58.0.

### Compound **3j**

GCMS: Calculated for C_11_H_14_NO_2_F_3_ M^+^ 250. Found: 250. ^1^H NMR (Acetone-d_6_, 300 MHz) *δ* 7.02 (m, 4H, aromatic), 4.07 (m, 2H, CH_2_), 2.61 (m, 2H, CH_2_), 2.51 (m, 4H, 2 × CH_2_), 1.9 (m, 2H, CH_2_), 0.99 (m, 6H, 2 × CH_3_); ^13^C NMR (Acetone-d_6_, 75.5 MHz) *δ* 158.1, 122.4, 116.1, 115.3, 66.3, 48.9, 46.8, 26.9, 11.5.

### Compound **3k**

GCMS: Calculated for C_14_H_20_NOCl M^+^ 242. Found: 242. ^1^H NMR (Acetone-d_6_, 300 MHz) *δ* 7.28 (d, J = 7.2 Hz, 2H, aromatic), 6.94 (d, J = 6.9 Hz, 2H, aromatic), 4.07 (t, J = 6.6 Hz, 2H, CH_2_), 2.66 (m, 8H, 4 × CH_2_), 1.07 (m, 6H, CH_3_); ^13^C NMR (Acetone-d_6_, 75.5 MHz) *δ* 129.1, 115.9, 46.7.

### Compound **5a**

GCMS: Calculated for C_15_H_23_NO M^+^ 234. Found: 234. ^1^H NMR (Acetone-d_6_, 300 MHz) *δ* 7.22 (m, 5 H, aromatic), 7.16 (d, J = 13.2 Hz, 1H), 5.86 (d, J = 12.9 Hz, 1H), 3.91 (t, J = 6.3 Hz, 2H, CH_2_), 2.49 (m, 6H, CH_2_), 1.79 (q, J = 7.2 Hz, 2H, CH_2_), 0.98 (t, J = 6.9 Hz, 6H, 2 × CH_3_); ^13^C NMR (Acetone-d_6_, 75.5 MHz) *δ* 148.4, 136.9, 132.7, 128.4, 124.8, 105.4, 67.9, 49.0, 46.7, 27.3, 11.6.

### Compound **5b**

GCMS: Calculated for C_15_H_22_NOF M^+^ 252. Found: 252. ^1^H NMR (Acetone-d_6_, 300 MHz) *δ* 7.62 − 7.01 (m, 5H, aromatic), 6.8 (d, 1H, CH), 5.90 (d, J = 12.9 Hz, 1H, CH), 3.94 (t, J = 6.6 Hz, 2H, CH_2_), 2.54 (m, 6H, 3 × CH_2_), 2.5 (t, 2H, CH_2_), 1.82 (m, 2H, CH_2_), 1.0 (t, J = 6.9 Hz, 6H, 2 × CH_3_); ^13^C NMR (Acetone-d_6_75.5 MHz) *δ* 149.3, 132.2, 129.0, 127.2, 116.2, 105.3, 68.9, 49.9, 47.7, 28.2, 12.5 ^19^F NMR (Acetone-d_6_, 300 MHz) *δ*–115.8, –119.5.

### Compound **5c**

GCMS: Calculated for C_16_H_25_NO M^+^ 247. Found: 247. ^1^H NMR (Acetone-d6, 300 MHz) *δ* 7.08 (m, 5H), 5.81 (d, J = 13.2 Hz, 1H, CH), 3.89 (t, J = 6.0 Hz, 2H, CH_2_), 2.48 (m, 6H, 3 × CH_2_), 2.25 (s, 3H, CH_3_), 1.78 (m, 2H, CH_2_), 0.98 (t, J = 6.9 Hz, 6H, 2 × CH_3_); ^13^C NMR (Acetone-d_6_, 75.5 MHz) *δ* 148.7, 135.5, 130.2, 127.1, 125.7, 106.3, 68.8, 50.0, 47.7, 28.2, 21.0, 12.5.

## Figures and Tables

**Table 1:** C–O bond by cross-coupling of potassium aryltrifluorobotates and hydroxyamines^a^.

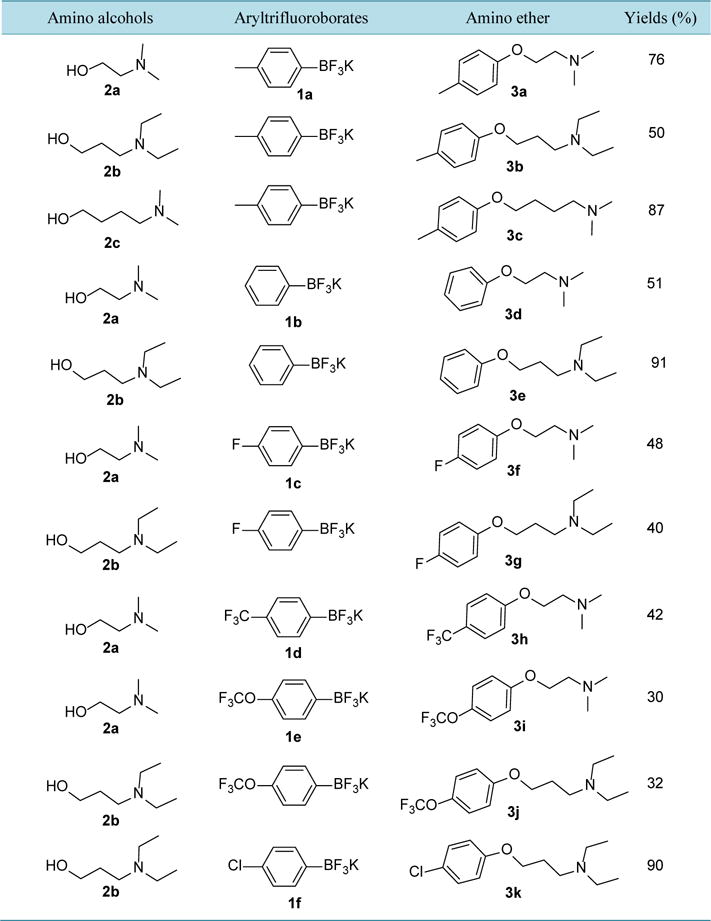

a Cu(OAc)_2_ (1.0 eq), ArBF_3_K **1** (2.5 eq), Hydroxylamine **2** (1.0 eq), K_2_CO_3_ (2.0 eq), 1,4-dioxane 2.0 mL *MW*, 140°C, 30 min.

**Table 2:** C–O bond by cross-coupling of potassium stryltrifluoroborates and hydroxyamines^a^.

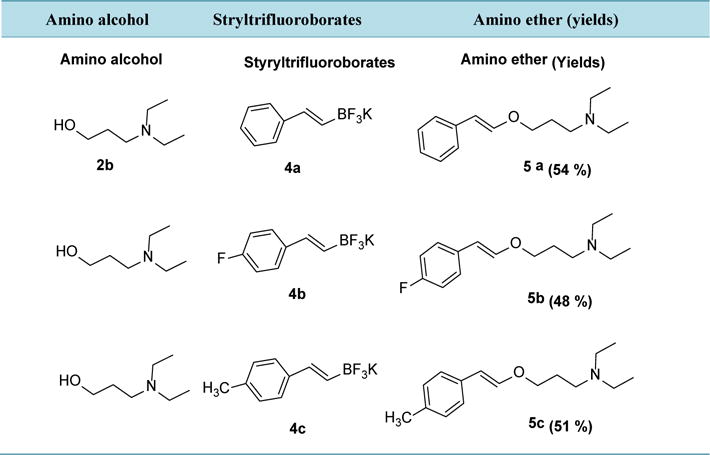

a Cu(OAc)_2_ (1.0 eq), StyrylBF_3_K **4** (2.5 eq), Hydroxylamine **2** (1.0 eq), K_2_CO_3_ (2.0 eq), 1,4-dioxane 2.0 mL *MW*, 140°C, 30 min.
